# Supplementary material for: Interleukin Gene Variability and Periodontal Bacteria in Patients with Generalized Aggressive Form of Periodontitis
Source: Int J Mol Sci. 2020 Jul 2;21(13):4728. doi: 10.3390/ijms21134728 (PMC7370291; doi:10.3390/ijms21134728)
Supplement: Supplementary file 1 [file ijms-21-04728-s001.pdf]

Table S1. The genetic association case-control studies and meta-analyses focused on polymorphisms in interleukin-1 (*IL-1*), *IL-6*, *IL-10*, *IL-17A*, and *IL-18* in patients with an aggressive form of periodontitis (AgP).

Gene	Polymorphism (rs number)	*N* (AgP/HC)	Form of AgP	Population	Association with AgP	Reference
IL-1A	–889 C/T (rs1800587)	91/210	Generalized	Czech	NS	Current study
		41+19/128	Generalized+localized	Algerian	T allele and TT genotype (risk)	[1]
		20/20	Generalized	Indian	TT genotype (risk)	[2]
		1266/2134 (meta-analysis including 19 studies)	Unknown	Mixed	NS	[3]
		80/80	Generalized	Iranian	NS	[4]
		55/41	Generalized	Brazilian	NS	[5]
IL-1B	+3953 (+3954) C/T (rs1143634)	91/210	Generalized	Czech	NS	Current study
		229/308 (meta-analysis including 3 studies)	Unknown	Mixed	NS	[6]
		43/50	Unknown	Brazilian	T allele in haplotype with *IL-1RN**S allele (risk)	[7]
		41+19/128	Generalized+localized	Algerian	T allele (risk)	[1]
		1594/2483 (meta-analysis including 25 studies)	Unknown	Mixed	NS	[8]
		26/26	Generalized	Iranian	TT genotype (risk)	[9]
		54/101	Unknown	Indian	NS	[10]
		28/33	Generalized	German	NS	[11]
IL-1RN	intron 2, 86 bp VNTR (rs2234663)	91/210	Generalized	Czech	NS	Current study
		43/50	Unknown	Brazilian	S allele, SS genotype, and haplotype with *IL-1B* +3953T allele (risk)	[7]
		577/904 (meta-analysis including 10 studies)	Unknown	Mixed (Caucasians 234/272)	NS (LS genotype protective for AgP in Caucasians)	[12]
		46/90	Generalized	Greek	NS	[13]
IL-6	–174 C/G (rs1800795)	91/210	Generalized	Czech	NS	Current study
		53/38	Generalized	Turkish	G allele and GG genotype (risk)	[14]
		53/50	Generalized	Turkish	G allele and GG genotype (risk)	[15]

		9+3/6	Generalized+localized	UK (white)	Haplotype was associated with inflammantory response in AgP patients	[16]
		122/246	Generalized	Italian	NS	[17]
		35/85	Generalized	Turkish	NS	[18]
		981/497 (meta-analysis including 6 studies)	Unknown (included also patients with CP)	Mixed (Caucasian 806/423)	G allele (risk)	[19]
		167 +57/231	Generalized+localized	UK (mixed)	NS	[20]
IL-10	–1087 (*–1082)* G/A (rs1800896)	91/210	Generalized	Czech	GG genotype (risk) GCC (rs1800896/ rs1800871/ rs1800872) haplotype (risk)	Current study
		1557/1447 (meta-analysis including 16 studies)	Unknown (included also patients with CP)	Mixed (non-Asian)	NS	[21]
		50/61	Generalized	Brazilian	NS	[22]
		85/86	Localized	Jordanian	NS	[23]
		197/502 (meta-analysis including 7 studies)	Unknown	Mixed (Caucasian 132/376)	NS	[24]
		122/246	Generalized	Italian	NS	[17]
		35/85	Generalized	Turkish	NS	[18]
		65/126	Generalized	Taiwan	Haplotype ATA/ATA (risk)	[25]
		55/43	Unknown	Brazilian	NS	[26]
		32/34	Generalized	German	Allele A (risk)	[27]
		52/61	Generalized	Iranian	NS	[28]
		51/100	Unknown	UK (white)	NS	[29]
	–829 (*–824)* C/T (rs1800871)	91/210	Generalized	Czech	GCC (rs1800896/ rs1800871/ rs1800872) haplotype (risk)	Current study
		1557/1447 (meta-analysis including 16 studies)	Unknown (included also patients with CP)	Mixed (non-Asian)	NS	[21]
		50/61	Generalized	Brazilian	NS	[22]
		97/269 (meta-analysis including 4 studies)	Unknown	Mixed (Caucasian 32/143)	NS	[24]
		35/85	Generalized	Turkish	NS	[18]
		65/126	Generalized	Taiwan	Haplotype ATA/ATA (risk)	[25]
		32/34	Generalized	German	Allele T (risk)	[27]

		6+12/21	Generalized+localized	German	NS	[30]
	–597 (*–592)* C/A (rs1800872)	91/210	Generalized	Czech	GCC (rs1800896/ rs1800871/ rs1800872) haplotype (risk)	Current study
		1557/1447 (meta-analysis including 16 studies)	Unknown (included also patients with CP)	Mixed	NS	[21]
		288/399 (meta-analysis including 6 studies)	Unknown	Mixed	Allele A and genotype AA (risk)	[31]
		53/38	Generalized	Turkish	Genotype AA	[14]
		53/50	Generalized	Turkish	Genotype AA risk	[15]
		85/86	Localized	Jordanian	NS	[23]
		97/442 (meta-analysis including 5 studies)	Unknown	Mixed (Caucasian 32/316)	NS	[24]
		122/246	Generalized	Italian	NS	[17]
		35/85	Generalized	Turkish	NS	[18]
		65/126	Generalized	Taiwan	Haplotype ATA/ATA (risk)	[25]
		32/34	Generalized	German	Allele A of haplotype ATA/ATA – present only in GAgP group	[27]
		6+12/21	Generalized+localized	German	NS	[30]
IL-17A	–197 A/G (rs2275913)	91/210	Generalized	Czech	NS	Current study
		188/655 (meta-analysis including 5 studies)	Unknown	Mixed	NS	[32]
		35/35	Localized	Indian	A allele and AA+AG genotypes risk	[33]
		45/72	Generalized+ localized	Brazilian	G allele and GG genotype associated with AgP in non-smokers	[34]
IL-18	–607 A/C (rs1946518)	91/210	Generalized	Czech	Haplotype AGC (rs1946518/rs187238/rs 4988359) (risk in dominant model)	Current study
		576/458 (meta-analysis including 5 studies)	Unknown (included also patients with CP)	Mixed	Only 1 study with AgP patients was included.	[35]
		109/100	Generalized	Italian	Genotype AA with CG genotype of -137	[36]

				(rs187238) associated with AgP	
	122/246	Generalized	Italian	NS	[17]
	111/80	Generalized	German	NS	[37]
	123/121	Unknown	German	NS	[38]
–137 C/G (rs187238)	91/210	Generalized	Czech	Haplotype AGC (rs1946518/rs187238/rs 4988359) (risk in dominant model)	Current study
	576/458 (meta-analysis including 5 studies)	Unknown (included also patients with CP)	Mixed	Only 1 study with AgP patients was included.	[35]
	109/100	Generalized	Italian	Genotype CG with AA genotype of -607 (rs1946518) associated with AgP	[36]
	111/80	Generalized	German	NS	[37]
	123/121	Unknown	German	NS	[38]

AgP, aggressive periodontitis; CP, chronic periodontitis; HC, healthy control; *N*, number; NS, nonsignificant; for references, see the end of this file.

References

1. Boukortt, K.N.; Saidi-Ouahrani, N.; Boukerzaza, B.; Ouhaibi-Djellouli, H.; Hachmaoui, K.; Benaissa, F.Z.; Taleb, L.; Drabla-Ouahrani, H.; Deba, T.; Ouledhamou, S.A.; et al. Association Analysis of the IL-1 Gene Cluster Polymorphisms with Aggressive and Chronic Periodontitis in the Algerian Population. *Arch. Oral Biol.* **2015**, *60*, 1463–1470, doi: 10.1016/j.archoralbio.2015.06.018.
2. Puri, K.; Chhokra, M.; Dodwad, V.; Puri, N. Association of Interleukin-1 α (–889) Gene Polymorphism in Patients with Generalized Aggressive and Chronic Periodontitis. *Dent. Res. J.* **2015**, *12*, 76–82, doi: 10.4103/1735-3327.150338.
3. Wang, W.-F.; Shi, J.; Chen, S.-J.; Niu, Y.-M.; Zeng, X.-T. Interleukin-1α -899 (+4845) C→T Polymorphism Is Not Associated with Aggressive Periodontitis Susceptibility: A Meta-analysis Based on 19 Case-control Studies. *Biomed. Rep.* **2014**, *2*, 378–383, doi: 10.3892/br.2014.240.
4. Kiani, Z.; Tavakkol-Afshari, J.; Hojjat, H.; Arab, H.R.; Radvar, M.; Sadeghizadeh, M.; Ebadian, A.R. Polymorphism of IL-1alpha(-889) Gene and Its Association with Aggressive Periodontitis. *Iran. J. Allergy Asthma Immunol.* **2009**, *8*, 95–98, doi.org/08.02/ijaai.9598.
5. Moreira, P.R.; Costa, J.E.; Gomez, R.S.; Gollob, K.J.; Dutra, W.O. The IL1A (-889) Gene Polymorphism Is Associated with Chronic Periodontal Disease in a Sample of Brazilian Individuals. *J. Periodontal Res.* **2007**, *42*, 23–30, doi.org/10.1111/j.1600-0765.2006.00910.x.
6. Huang, W.; He, B.-Y.; Shao, J.; Jia, X.-W.; Yuan, Y.-D. Interleukin-1β Rs1143627 Polymorphism with Susceptibility to Periodontal Disease. *Oncotarget* **2017**, *8*, 31406–31414, doi: 10.18632/oncotarget.15612.
7. Ribeiro, M.S.M.; Pacheco, R.B.A.; Fischer, R.G.; Macedo, J.M.B. Interaction of IL1B and IL1RN Polymorphisms, Smoking Habit, Gender, and Ethnicity with Aggressive and Chronic Periodontitis Susceptibility. *Contemp. Clin. Dent.* **2016**, *7*, 349, doi: 10.4103/0976-237X.188560.
8. Chen, Y.-J.; Han, Y.; Mao, M.; Tan, Y.-Q.; Leng, W.-D.; Zeng, X.-T. Interleukin-1β Rs1143634 Polymorphism and Aggressive Periodontitis Susceptibility: A Meta-Analysis. *Int. J. Clin. Exp. Med.* **2015**, *8*, 2308–2316.
9. Ayazi, G.; Pirayesh, M.; Yari, K. Analysis of Interleukin-1β Gene Polymorphism and Its Association with Generalized Aggressive Periodontitis Disease. *DNA Cell Biol.* **2013**, *32*, 409–413, doi: 10.1089/dna.2012.1905
10. Shete, A.R.; Joseph, R.; Vijayan, N.N.; Srinivas, L.; Banerjee, M. Association of Single Nucleotide Gene Polymorphism at Interleukin-1β +3954, −511, and −31 in Chronic Periodontitis and Aggressive Periodontitis in Dravidian Ethnicity. *J. Periodontol.* **2010**, *81*, 62–69, doi.org/10.1902/jop.2009.090256.

11. Gonzales, J.R.; Michel, J.; Rodriguez, E.L.; Herrmann, J.M.; Bodeker, R.H.; Meyle, J. Comparison of Interleukin-1 Genotypes in Two Populations with Aggressive Periodontitis. *Eur. J. Oral Sci.* **2003**, *111*, 395–399, doi.org/10.1034/j.1600-0722.2003.00071.x
12. Ding, C.; Zhao, L.; Sun, Y.; Li, L.; Xu, Y. Interleukin-1 Receptor Antagonist Polymorphism (Rs2234663) and Periodontitis Susceptibility: A Meta-Analysis. *Arch. Oral Biol.* **2012**, *57*, 585–593, doi: 10.1016/j.archoralbio.2012.01.016.
13. Sakellari, D.; Katsares, V.; Georgiadou, M.; Kouvatsi, A.; Arsenakis, M.; Konstantinidis, A. No Correlation of Five Gene Polymorphisms with Periodontal Conditions in a Greek Population. *J. Clin. Periodontol.* **2006**, *33*, 765–770, doi.org/10.1111/j.1600-051X.2006.00983.x
14. Toker, H.; Görgün, E.P.; Korkmaz, E.M. Analysis of IL-6, IL-10 and NF-KB Gene Polymorphisms in Aggressive and Chronic Periodontitis. *Cent. Eur. J. Public Health* **2017**, *25*, 157–162, doi.org/10.21101/cejph.a4656.
15. Pirim Gorgun, E.; Toker, H.; Korkmaz, E.M.; Poyraz, O. IL-6 and IL-10 Gene Polymorphisms in Patients with Aggressive Periodontitis: Effects on GCF, Serum and Clinic Parameters. *Braz. Oral Res.* **2017**, *31*, e12. doi: 10.1590/1807-3107BOR-2017.vol31.0012.
16. Nibali, L.; Pelekos, G.; D'Aiuto, F.; Chaudhary, N.; Habeeb, R.; Ready, D.; Parkar, M.; Donos, N. Influence of IL-6 Haplotypes on Clinical and Inflammatory Response in Aggressive Periodontitis. *Clin. Oral Investig.* **2013**, *17*, 1235–1242, doi: 10.1007/s00784-012-0804-3.
17. Scapoli, C.; Mamolini, E.; Carrieri, A.; Guarnelli, M.E.; Annunziata, M.; Guida, L.; Romano, F.; Aimetti, M.; Trombelli, L. Gene—gene interaction among cytokine polymorphisms influence susceptibility to aggressive periodontitis. *Genes Immun*. **2011**, *12*, 473–480, doi: /10.1038/gene.2011.28.
18. Erciyas, K.; Pehlivan, S.; Sever, T.; Igci, M.; Arslan, A.; Orbak, R. Association between TNF-α, TGF-B1, IL-10, IL-6 and IFN-γ Gene Polymorphisms and Generalized Aggressive Periodontitis. *Clin. Invest. Med.* **2010**, *33*, E85–E91. doi: 10.25011/cim.v33i2.12346.
19. Shao, M.; Huang, P.; Cheng, R.; Hu, T. Interleukin-6 Polymorphisms Modify the Risk of Periodontitis: A Systematic Review and Meta-Analysis. *J. Zhejiang Univ. Sci. B* **2009**, *10*, 920–927, doi: 10.1631/jzus.B0920279.
20. Nibali, L.; Donos, N.; Brett, P.M.; Parkar, M.; Ellinas, T.; Llorente, M.; Griffiths, G. S. A Familial Analysis of Aggressive Periodontitis – Clinical and Genetic Findings. *J. Periodontal Res.* **2008**, *43*, 627–634, doi: 10.1111/j.1600-0765.2007.01039.x.
21. Wong, H.C.; Ooi, Y.; Pulikkotil, S.J.; Naing, C. The Role of Three Interleukin 10 Gene Polymorphisms (− 1087 A > G, − 824 C > T, − 597 A > C) in the Risk of Chronic and Aggressive Periodontitis: A Meta-Analysis and Trial Sequential Analysis. *BMC Oral Health* **2018**, *18*, 171, doi: 10.1186/s12903-018-0637-9.
22. Silveira, V.R.S.; Pigossi, S.C.; Scarel-Caminaga, R.M.; Cirelli, J.A.; Rêgo, R.; Nogueira, N.A.P. Analysis of Polymorphisms in Interleukin 10, NOS2A, and ESR2 Genes in Chronic and Aggressive Periodontitis. *Braz. Oral Res.* **2016**, *30*, doi.org/10.1590/1807-3107BOR-2016.vol30.0105
23. Jaradat, S.M.; Ababneh, K.T.; Jaradat, S.A.; Abbadi, M.S.; Taha, A.H.; Karasneh, J.A.; Haddad, H.I. Association of Interleukin-10 Gene Promoter Polymorphisms with Chronic and Aggressive Periodontitis. *Oral Dis*. **2012**, *18*, 271–279, doi: 10.1111/j.1601-0825.2011.01872.x.
24. Albuquerque, C.M.; Cortinhas, A.J.; Morinha, F.J.; Leitão, J.C.; Viegas, C.A.; Bastos, E.M. Association of the IL-10 Polymorphisms and Periodontitis: A Meta-Analysis. *Mol. Biol. Rep.* **2012**, *39*, 9319–9329, doi: 10.1007/s11033-012-1738-1.
25. Hu, K.-F.; Huang, K.-C.; Ho, Y.-P.; Lin, Y.-C.; Ho, K.-Y.; Wu, Y.-M.; Yang, Y.-H.; Tsai, C.-C. Interleukin-10 (−592 C/A) and Interleukin-12B (+16974 A/C) Gene Polymorphisms and the Interleukin-10 ATA Haplotype Are Associated with Periodontitis in a Taiwanese Population. *J. Periodontal Res.* **2009**, *44*, 378–385, doi.org/10.1111/j.1600-0765.2008.01116.x
26. Moreira, P. R.; Costa, J. E.; Gomez, R. S.; Gollob, K. J.; Dutra, W. O. TNFA and IL10 Gene Polymorphisms Are Not Associated with Periodontitis in Brazilians. *Open Dent. J.* **2009**, *3*, 184–190. doi.org/10.2174/1874210600903010184.
27. Reichert, S.; Machulla, H.K.G.; Klapproth, J.; Zimmermann, U.; Reichert, Y.; Gläser, C.H.; Schaller, H.G.; Stein, J.; Schulz, S. The Interleukin-10 Promoter Haplotype ATA Is a Putative Risk Factor for Aggressive Periodontitis. *J. Periodontal Res.* **2008**, *43*, 40–47, doi: 10.1111/j.1600-0765.2007.00992.x.
28. Mellati, E.; Arab, H.R.; Tavakkol-Afshari, J.; Ebadian, A.R.; Radvar, M. Analysis of -1082 IL-10 Gene Polymorphism in Iranian Patients with Generalized Aggressive Periodontitis. *Med. Sci. Monit.* **2007**, *13*, CR510–CR514.

29. Brett, P.M.; Zygogianni, P.; Griffiths, G.S.; Tomaz, M.; Parkar, M.; D'Aiuto, F.; Tonetti, M. Functional Gene Polymorphisms in Aggressive and Chronic Periodontitis. *J. Dent. Res.* **2005**, *84,* 1149–1153, doi: 10.1177/154405910508401211.
30. Gonzales, J.R.; Michel, J.; Diete, A.; Herrmann, J.M.; Bödeker, R.H.; Meyle, J. Analysis of Genetic Polymorphisms at the Interleukin-10 Loci in Aggressive and Chronic Periodontitis. *J. Clin. Periodontol.* **2002**, *29*, 816–822, doi.org/10.1034/j.1600-051X.2002.290905.x
31. Li, Y.; Feng, G.; Deng, Y.; Song, J. Contribution of Interleukin-10-597 (-590, -597) C>A Polymorphisms to Periodontitis Susceptibility: An Updated Meta-Analysis Based on 18 Case-Control Studies. *Dis. Markers* **2018**, *2018*, 2645963, doi: 10.1155/2018/2645963.
32. da Silva, F.R.P.; Pessoa, L. dos S.; Vasconcelos, A.C.C.G.; de Aquino Lima, W.; Alves, E.H.P.; Vasconcelos, D.F.P. Polymorphisms in Interleukins 17A and 17F Genes and Periodontitis: Results from a Meta-Analysis. *Mol. Biol. Rep.* **2017**, *44*, 443–453, doi: 10.1007/s11033-017-4128-x.
33. Chaudhari, H.L.; Warad, S.; Ashok, N.; Baroudi, K.; Tarakji, B. Association of Interleukin-17 Polymorphism (-197G/A) in Chronic and Localized Aggressive Periodontitis. *Braz. Oral Res.* **2016**, *30*, pii: S1806-83242016000100219, doi: 10.1590/1807-3107BOR-2016.vol30.0026
34. Saraiva, A.M.; Alves e Silva, M.R.M.; Correia Silva, J. de F.; da Costa, J.E.; Gollob, K.J.; Dutra, W.O.; Moreira, P.R. Evaluation of IL17A Expression and of IL17A, IL17F and IL23R Gene Polymorphisms in Brazilian Individuals with Periodontitis. *Hum. Immunol.* **2013**, *74*, 207–214, doi: 10.1016/j.humimm.2012.10.026.
35. Li, Z.-G.; Li, J.-J.; Sun, C.-A.; Jin, Y.; Wu, W.-W. Interleukin-18 Promoter Polymorphisms and Plasma Levels Are Associated with Increased Risk of Periodontitis: A Meta-Analysis. *Inflamm. Res.* **2014**, *63*, 45–52, doi: 10.1007/s00011-013-0669-1.
36. Martelli, F.S.; Mengoni, A.; Martelli, M.; Rosati, C.; Fanti, E. IL-18 Gene Promoter Polymorphisms Are Only Moderately Associated with Periodontal Disease in Italian Population. *Clin. Cases Miner. Bone Metab.* **2012**, *9*, 153–156.
37. Noack, B.; Görgens, H.; Lorenz, K.; Ziegler, A.; Hoffmann, T.; Schackert, H. K. TLR4 and IL-18 Gene Variants in Aggressive Periodontitis. *J. Clin. Periodontol.* **2008**, *35*, 1020–1026, doi: 10.1111/j.1600-051X.2008.01334.x
38. Folwaczny, M.; Glas, J.; Török, H.-P.; Tonenchi, L.; Paschos, E.; Bauer, B.; Limbersky, O.; Folwaczny, C. Polymorphisms of the Interleukin-18 Gene in Periodontitis Patients. *J. Clin. Periodontol.* **2005**, *32*, 530–534, doi: 10.1111/j.1600-051X.2005.00711.x
